# Role of central vagal 5-HT_3_ receptors in gastrointestinal physiology and pathophysiology

**DOI:** 10.3389/fnins.2015.00413

**Published:** 2015-10-29

**Authors:** Kirsteen N. Browning

**Affiliations:** Department of Neural and Behavioral Sciences, Penn State University College of MedicineHershey, PA, USA

**Keywords:** vagus, vagal afferent, 5-HT, plasticity, gastrointestinal

## Abstract

Vagal neurocircuits are vitally important in the co-ordination and modulation of GI reflexes and homeostatic functions. 5-hydroxytryptamine (5-HT; serotonin) is critically important in the regulation of several of these autonomic gastrointestinal (GI) functions including motility, secretion and visceral sensitivity. While several 5-HT receptors are involved in these physiological responses, the ligand-gated 5-HT_3_ receptor appears intimately involved in gut-brain signaling, particularly via the afferent (sensory) vagus nerve. 5-HT is released from enterochromaffin cells in response to mechanical or chemical stimulation of the GI tract which leads to activation of 5-HT_3_ receptors on the terminals of vagal afferents. 5-HT_3_ receptors are also present on the soma of vagal afferent neurons, including GI vagal afferent neurons, where they can be activated by circulating 5-HT. The central terminals of vagal afferents also exhibit 5-HT_3_ receptors that function to increase glutamatergic synaptic transmission to second order neurons of the nucleus tractus solitarius within the brainstem. While activation of central brainstem 5-HT_3_ receptors modulates visceral functions, it is still unclear whether central vagal neurons, i.e., nucleus of the tractus solitarius (NTS) and dorsal motor nucleus of the vagus (DMV) neurons themselves also display functional 5-HT_3_ receptors. Thus, activation of 5-HT_3_ receptors may modulate the excitability and activity of gastrointestinal vagal afferents at multiple sites and may be involved in several physiological and pathophysiological conditions, including distention- and chemical-evoked vagal reflexes, nausea, and vomiting, as well as visceral hypersensitivity.

## Vago-vagal reflex control of GI tract

Despite intrinsic (enteric) neural plexuses that allow a considerable degree of autonomy over digestive functions, the central nervous system (CNS) provides extrinsic neural inputs to the GI tract that govern, regulate and modulate these functions. The GI tract receives extrinsic neural inputs from both parasympathetic and sympathetic pathways derived (or controlled) from caudal brainstem nuclei (Browning and Travagli, 2014). While the sympathetic nervous system exerts a predominantly inhibitory effect upon GI muscle and mucosal secretion and regulates GI blood flow via neurally-dependent vasoconstriction, the parasympathetic nervous system exerts both excitatory and inhibitory control over gastric and intestinal motility and tone suggesting a more finely tuned regulation of GI functions (Travagli et al., 2006). The esophagus, stomach, and upper GI tract, in particular, receive a dense parasympathetic innervation, the intensity of which decreases as one progresses distally through the intestine (Berthoud et al., 1991).

The parasympathetic innervation to the stomach, small intestine and proximal colon is provided by the vagus nerve. A mixed nerve, containing both sensory and motor fibers, the vagus contains approximately 70–80% sensory fibers that transduce physiological events within the GI tract and relay this information to the CNS. Anatomical and physiological studies have characterized several different types of vagal afferent fibers that can be distinguished based upon their responses to muscle tension or pressure (mostly low-threshold mechanosensors although high-threshold nociceptors are also present), the location of the afferent fibers receptive field (muscle, mucosal, or serosal/mesenteric) and their principle stimulus modality (chemical, osmotic, mechanical) as well as the region of the GI tract they innervate (Powley and Phillips, 2002; Beyak and Grundy, 2005).

**Figure 1:**
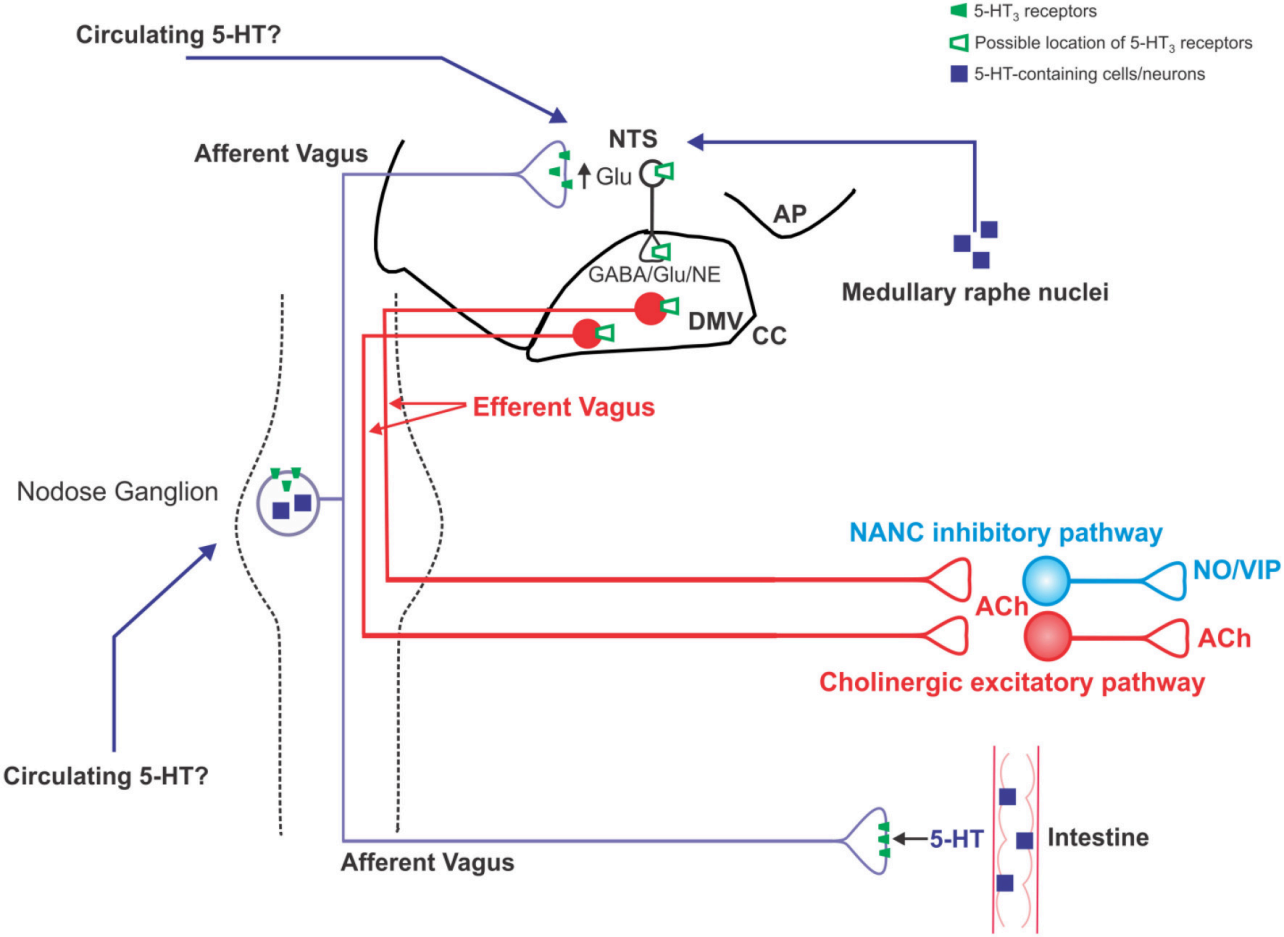
**Schematic illustration of the location of 5-HT_3_ receptors on vagal neurocircuits**. 5-HT is released from intestinal enteroendocrine cells in response to ingested carbohydrates and acts locally on 5-HT_3_ receptors present on vagal afferent peripheral terminals to increase vagal afferent fiber firing. Circulating 5-HT may also modulate vagal afferent fiber excitability via actions at 5-HT_3_ receptors on the soma of subpopulations of nodose ganglion neurons, as well as the central terminals of vagal afferent fibers within the brainstem. Some nodose ganglion neurons are themselves serotonergic, although it is unclear whether they release 5-HT in a physiologically-relevant manner; serotonergic medullary raphe neurons are an additional potential source of 5-HT input into vagal brainstem neurocircuits. An increase in vagal afferent fiber excitability, as results from activation of 5-HT_3_ receptors, increase glutamatergic transmission to second order NTS neurons. It is unclear whether NTS and DMV neurons themselves display functional 5-HT_3_ receptors or whether the observed alterations in their activity is subsequent to the modulation of vagal afferent fiber neurotransmission.

The cell bodies of vagal sensory afferents, which lie within the paired nodose ganglia or nodose-jugular complex, serve the classic afferent functions and relay the peripheral sensory information from the GI tract to the brainstem via a glutamatergic synapse at the level of the nucleus tractus solitarius (NTS). NTS neurons assimilate this enormous volume of sensory information and integrate it with inputs received from other brainstem and higher CNS centers involved in autonomic homeostatic functions. Indeed, the NTS has either reciprocal connections with, or receives inputs from, the hypothalamus, amygdala, nucleus accumbens, raphe, trigeminal, vestibular, and parabrachial nuclei as well as the area postrema, all of which help to sculpt and shape these vagal afferent visceral sensory inputs. The integrated signal is then relayed from the NTS to the adjacent dorsal motor nucleus of the vagus (DMV) which contains the preganglionic parasympathetic motorneurons which supply the parasympathetic output to the upper GI tract via the efferent vagus nerve (Figure 1; Travagli et al., 2006; Browning and Travagli, 2014).

## 5-HT_3_ receptors and vagal sensory functions

5-HT is an important neurotransmitter in several GI functions, and >90% of the total body 5-HT is contained with the GI tract, either within specialized enteroendocrine cells, termed enterochromaffin (EC) cells or within neurons. Excellent recent reviews have provided in depth coverage of the role of 5-HT within the GI tract (Gershon and Tack, 2007; Mawe and Hoffman, 2013); this review, therefore, will concentrate on the role of 5-HT_3_ receptors in gut-brain and brain-gut signaling outside the GI tract itself. Electrophysiological studies have demonstrated functionally active 5-HT_3_ receptors on vagal afferent neurons and fibers (Leal-Cardoso et al., 1993; Hillsley et al., 1998; Kreis et al., 2002; Moore et al., 2002; Lacolley et al., 2006a; Babic et al., 2012) and activation of 5-HT_3_ receptors induces a short latency, transient increase in firing rate of vagal afferents (Hillsley and Grundy, 1998; Hillsley et al., 1998) or a brief, rapid inward current (or membrane depolarization), in isolated neurons (Leal-Cardoso et al., 1993; Peters et al., 1993; Babic et al., 2012) consistent with its function as a ligand-gated cation channel (Derkach et al., 1989).

When released from EC cells, 5-HT triggers smooth muscle activity via activation of 5-HT_3_ receptors on intrinsic primary afferent neurons (IPANs; Tuladhar et al., 1997; Zhou and Galligan, 1999; Bertrand et al., 2000; Gwynne and Bornstein, 2007). Such motor responses can, and do, activate extrinsic vagal and spinal afferent fibers, possibly via 5-HT_2_ receptors secondary to smooth muscle activity (Blackshaw and Grundy, 1993; Hillsley and Grundy, 1998; Hillsley et al., 1998). The released 5-HT also activates extrinsic primary afferent terminals directly, however, via activation of 5-HT_3_ receptors (Paintal, 1951; Hillsley et al., 1998). EC cells release 5-HT in response to mechanical (Bulbring and Lin, 1958; Blackshaw and Grundy, 1993; Mazda et al., 2004) as well as chemical stimulation. Luminal micronutrient content, in particular carbohydrates and hyperosmotic stimuli, induce strongly the release of 5-HT (Raybould and Zittel, 1995; Zhu et al., 2001; Raybould et al., 2003; Wu et al., 2005). Vagal afferent nerve terminals innervate the apical tips of mucosal villi as well as intestinal crypts and are likely, therefore, to be in close apposition to GI neurohormones, including 5-HT, released from mucosal enteroendocrine cells (Powley et al., 2011). While a large proportion of the 5-HT from EC cells may be released in close proximity to 5-HT_3_-containing primary afferent terminals, a significant amount is still absorbed into the bloodstream, and circulating platelet-free 5-HT levels rise almost three-fold following a meal (Houghton et al., 2003). In this regard, it is important to note that (1) 5-HT_3_ receptors are also present on nodose neuronal membranes (Leal-Cardoso et al., 1993; Moore et al., 1999, 2002; Lacolley et al., 2006a), including those innervating the GI tract (Daly et al., 2011; Babic et al., 2012), (2) 5-HT_3_ receptors are also present on the central terminals of vagal afferents within the brainstem (Glaum et al., 1992; Ramage and Mifflin, 1998; Wan and Browning, 2008b; Takenaka et al., 2011; Cui et al., 2012; Hosford et al., 2014), and (3) circulating mediators have far freer access to vagal soma and the brainstem than perhaps thought previously (Figure 1; Lacolley et al., 2006b; Baptista et al., 2007). This suggests that EC-derived circulating 5-HT has the potential to modulate vagal afferent neuronal activity at sites distinct from the GI tract and may, therefore, prolong or amplify local GI signaling.

Within the brainstem, activation of 5-HT_3_ receptors on vagal afferent terminals increases glutamatergic transmission to second order NTS neurons causing their activation (Glaum et al., 1992; Jeggo et al., 2005; Wan and Browning, 2008b; Takenaka et al., 2011; Cui et al., 2012; Hosford et al., 2014). NTS neurons are critically important in the regulation and modulation of a wide variety of autonomic homeostatic functions including cardiovascular as well as gastrointestinal processes (Andresen and Kunze, 1994; Travagli et al., 2006). Activation of vagal 5-HT_3_ receptors has been shown to be important in in baroreceptor and chemoreceptor reflex control of the cardiovascular system (Sévoz et al., 1996, 1997; Callera et al., 1997; Jeggo et al., 2005; Jordan, 2005; Ramage and Villalon, 2008) as well as pancreatic secretion (Mussa et al., 2008, 2010), meal termination, early satiety, and appetite regulation (Hayes and Covasa, 2006a; Wu et al., 2012).

The source of 5-HT activating 5-HT_3_ receptors on the central terminals of vagal afferents is the subject of some debate. 5-HT_3_ receptor selective antagonists decrease glutamatergic synaptic transmission from central vagal afferent terminals (Wan and Browning, 2008b; Cui et al., 2012; Hosford et al., 2014) suggesting the receptors are active tonically, although other studies have not observed this ongoing receptor activation (Cui et al., 2012). Such disparities may be explained by either experimental differences, since tonic 5-HT_3_ receptor activation was noted in studies employing coronal rather than horizontal brainstem slices, or species differences, being noted in studies involving rats, rather than mice. Immunohistochemical studies have demonstrated a dense serotonergic input into the dorsal vagal complex (i.e., NTS, DMV, and area postrema from the raphe nuclei (Steinbusch, 1981; Thor and Helke, 1987, 1989) the projections of which are more likely to remain intact in the coronal plane. It should also be noted, however, that the dorsal vagal complex is essentially a circumventricular organ with fenestrated capillaries and a leaky blood brain barrier (Cottrell and Ferguson, 2004; Fry and Ferguson, 2007) and circulating neurohormones or neuromodulators may have freer access to neurons within these areas (Baptista et al., 2007). It remains to be determined, however, whether elevations in circulating platelet-free 5-HT levels that occur in response to meal ingestion or mechanical stimulation exert any modulatory role on central vagal afferent neurotransmission. It should also be noted, however, that a subpopulation of nodose ganglion neurons have been shown to synthesize 5-HT (Gaudin-Chazal et al., 1982; Thor et al., 1988; Nosjean et al., 1990), although it is unclear whether vagal afferents are able to release 5-HT centrally under physiological conditions.

## 5-HT_3_ receptors and vagal motor functions

Surprisingly, it is not clear whether NTS and DMV neurons themselves display functional 5-HT_3_ receptors. Extracellular brainstem recordings have certainly demonstrated an alteration in NTS and DMV neuronal activity in response to both peripheral and central administration of 5-HT_3_ receptor agonists (Wang et al., 1996; Pires et al., 1998; Jeggo et al., 2005; Ramage and Villalon, 2008) while nerve recordings have demonstrated that vagal efferent activity is modulated following activation of 5-HT_3_ receptors (Mussa et al., 2010). The location of these 5-HT_3_ receptors has not been elucidated precisely; electron microscopy has shown that 5-HT_3_ receptors are present on neurons and glial cells within the brainstem suggesting an involvement in modulating postsynaptic neuronal responses as well presynaptic neurotransmitter release (Huang et al., 2004). Indeed, one relatively early study (Glaum et al., 1992) demonstrated that NTS neurons were depolarized by exogenous application of a 5-HT_3_ receptor agonist in a manner resistant to synaptic blockade, suggesting a postsynaptic receptor location. The alteration in neuronal activity in the majority of the remaining studies, however, could conceivably be the downstream response following increased glutamate release subsequent to activation of vagal afferent terminal 5-HT_3_ receptors.

## Physiological roles of vagal 5-HT_3_ receptor signaling

The physiological, rather than pathophysiological, role of vagal afferent 5-HT_3_ receptors following GI-mechanical or distention-related 5-HT release appears to still be open to debate. Several studies have demonstrated that mechanical stimulation of the GI tract activates vagal afferents; some studies describe this as direct activation of peripheral primary afferent 5-HT_3_ receptors (Mazda et al., 2004; Hayes and Covasa, 2006b), while others show this clearly to be an indirect effect, secondary to stimulation of local motor activity in response to the released 5-HT (Blackshaw and Grundy, 1993; Hillsley and Grundy, 1998; Hillsley et al., 1998). Indeed, recent work has suggested that while release of 5-HT from intestinal EC cells may not be a requirement for either the initiation or propagation of colonic motor complexes, 5-HT certainly modulates these peristaltic reflexes in a manner that appears to involve 5-HT_3_ receptors (Keating and Spencer, 2010; Spencer et al., 2011).

In contrast, chemically-stimulated 5-HT release has well-defined actions to activate vagal afferent 5-HT_3_ receptors directly. Ingestion of carbohydrates such as glucose, for example, induces a vagally-dependent gastric relaxation and delay in gastric emptying that is dependent upon peripheral vagal afferent 5-HT_3_ receptor activation; furthermore, peripheral application of 5-HT_3_ receptor selective agonists decrease gastric motility and delay gastric emptying (MacGregor et al., 1976; Rayner et al., 2001; Zhu et al., 2001; Raybould et al., 2003). Indeed, peripheral vagal afferent 5-HT_3_ receptor activation appears to play an ongoing modulatory role in the regulation of gastric motility and emptying since administration of 5-HT_3_ receptor selective antagonists accelerates gastric transit, suggesting the receptors may be under some degree of tonic activation (Coleman et al., 2003; Raybould et al., 2003; Gentilcore et al., 2007). The physiological role that 5-HT_3_ receptors on the central terminals of vagal afferents plays in the glucose-induced, vagally-dependent decrease in gastric motility and tone has still to be elucidated. Studies have demonstrated, however, that the response of vagal afferents to ingested glucose can be modulated by intravenous glucose (Mei, 1978) implying that glucose is capable of modulating vagal activity at sites other than afferent terminals within the GI tract. Indeed, studies have shown that a some GI-vagal afferent neurons are glucose-sensitive, that is, glucose can modulate the excitability of a subpopulation of GI nodose ganglion neurons via actions at ATP-sensitive potassium channels, in a manner similar to the canonical model of pancreatic β–cells (Grabauskas et al., 2010). This implies that, in addition to increasing vagal afferent activity via 5-HT release and subsequent 5-HT_3_ receptor activation, once absorbed form the GI tract, circulating glucose may also regulate nodose neuron excitability to modify the increase in vagal activity induced by luminal glucose. In addition to these actions of glucose, however, we have demonstrated that extracellular glucose levels are also able to modulate the density and function of 5-HT_3_ receptors on GI nodose neurons. In particular, increasing extracellular glucose levels induces the trafficking of existing 5-HT_3_ receptors to the membrane of GI-projecting vagal afferent neurons and increases the magnitude of the 5-HT-induced inward current, whereas decreasing glucose levels induce 5-HT_3_ receptor internalization and decrease the 5-HT-dependent inward current (Babic et al., 2012). Thus, ingested glucose may be able to amplify and prolong its afferent signaling by first releasing 5-HT from intestinal EC cells, and then by increasing the number of 5-HT_3_ receptors on vagal afferents available for activation.

The glucose-dependent modulation of 5-HT_3_ receptor trafficking and function also appears to occur centrally. We, and others, have demonstrated that extracellular glucose regulates the density of 5-HT_3_ receptors on vagal afferent central terminals; elevating extracellular glucose increases spontaneous and evoked glutamate release from vagal afferent terminals via actions in a 5-HT_3_ receptor-dependent manner (Wan and Browning, 2008a; Hosford et al., 2014) although the role of vagal afferent 5-HT_3_ receptors in the glucose-dependent modulation of gastric functions remains to be defined. Similarly, the concentration of glucose within the NTS parenchyma, and fluctuations in response to alterations in circulating glycemic levels, remain to be determined but concentrations within the cerebrospinal fluid are typically two-thirds those of circulating levels. As discussed previously, the dorsal vagal complex is a circumventricular organ and NTS neurons and fiber terminals may well be exposed to higher glucose levels than those measured elsewhere within the CNS (Dunn-Meynell et al., 2009). While the majority of electrophysiological studies in brainstem slice preparations certainly use non-physiological levels of glucose, we have demonstrated previously that glucose modulates glutamate release from vagal afferent terminals at much lower levels of extracellular glucose (0.5–5 mM; Browning, 2013) implying this is a physiological, rather than pathophysiological, phenomenon.

## Pathophysiological roles of vagal 5-HT_3_ receptor signaling

The sensory vagus nerve is generally considered to relay predominately non-noxious, interoceptive information from the GI tract to the brainstem although growing evidence suggests the involvement of the vagus nerve in pain processing (see Randich and Gebhart, 1992) Certainly, some vagal afferent fibers appear responsive to nociceptive stimulation although the primary response to noxious vagal afferent stimulation may be nausea, rather than pain (Chen et al., 2008).

Vagal neurocircuits have a well-described role in nausea and vomiting (see Babic and Browning, 2014) and the role of vagal afferent fibers in emesis have been most extensively studied in the context of chemotherapy-induced nausea and vomiting (CINV) or postoperative nausea and vomiting (PONV). Several chemotherapy agents induce the release of 5-HT from EC cells which activates 5-HT_3_ receptors on vagal afferent terminals (Endo et al., 1990, 2000; Horn et al., 2004); vagotomy decreases emesis induced by cytotoxic drugs while 5-HT_3_ receptor selective antagonists are particularly efficacious clinically in preventing CINV and PONV (Hawthorn et al., 1988; Andrews et al., 1990; Endo et al., 2000; Darmani and Johnson, 2004; Andrews and Horn, 2006). The presumed site of action of these 5-HT_3_ receptor selective antagonists is at peripheral vagal afferent terminals (Endo et al., 2000) although it should be noted that centrally applied 5-HT_3_ receptor antagonists also attenuate CINV, suggesting actions at brainstem 5-HT_3_ receptors (Leslie et al., 1990; Reynolds et al., 1991; Liu et al., 2003; Darmani and Ray, 2009) while 5-HT-induced disruptions in normal GI motility patterns may also contribute to CINV and PONV (Endo et al., 2000; Glatzle et al., 2002; Tonini, 2005). Similarly, the nausea and vomiting associated with several infectious agents, including rotavirus (Hagbom et al., 2011), *Salmonella typhimurium* (Jensen et al., 1997), and campylobacter (Blakelock and Beasley, 2003) has also been associated with the activation of vagal afferent 5-HT_3_ receptors subsequent to intestinal 5-HT release.

The role of vagal afferent 5-HT_3_ receptors in various forms of visceral hypersensitivity and nociceptive processing has been the focus of considerable attention from several groups although there are conflicting reports as to the extent of the involvement of vagal, rather than spinal, pathways. Several studies have suggested that vagal afferent fibers, and vagal afferent 5-HT_3_ receptors in particular, are important in the inhibitory modulation of spinal nociceptive transmission. Briefly, when administered intravenously, 5-HT induces a dose-dependent inhibition of the tail flick reflex, and this anti-nociceptive effect is dependent upon intact vagal pathways since it is abolished by either cervical vagotomy, nodose ganglionectomy, or neonatal capsaicin pretreatment (Meller et al., 1992). In a similar manner, vagotomized rats display an enhanced visceromotor response to colorectal distention (allodynia), effects that are lost following application of the local anesthetic lidocaine to the abdominal vagus (Chen et al., 2008). The specific involvement of 5-HT_3_ receptors in these responses was confirmed by studies investigating stress-induced visceral hyperalgesia, which demonstrated that subcutaneous administration of 5-HT_3_ receptor selective antagonists increased the visceromotor response to colorectal distension, actions that were prevented by perivagal capsaicin (Bradesi et al., 2007, NB—it should be noted that perivagal capsaicin does not produce a selective vagal deafferentation but also causes a significant degree of damage to vagal efferent motoneurons, Browning et al., 2013a). Thus, it appears that 5-HT_3_ receptor-dependent activation of vagal afferents inhibits the noxious stimulation of spinal afferents although the central nuclei responsible for this descending modulation have not been defined fully (Ren et al., 1990; Randich and Gebhart, 1992).

Such an anti-nociceptive role of vagal afferent 5-HT_3_ receptors appears consistent across several visceral hypersensitivity models suggesting common mechanistic pathophysiologies. In experimental models of duodenal acidification-induced gastric hypersensitivity, for example, intestinal acidification enhances the pressor response observed in response to gastric distention; this pressor response is enhanced by 5-HT_3_ receptor selective agonists (Nakata-Fukuda et al., 2014) while administration of 5-HT_3_ receptor selective antagonists inhibits the sensitization to distention that occurs in humans (Vanuytsel et al., 2011).

Activation of vagal afferent 5-HT_3_ receptors also have well described roles in the immune responses elicited by antigen challenge in sensitized animal models, where 5-HT released following mast cell degranulation activates vagal afferents to modulate the visceral hypersensitivity and motor response to the immune challenge (Castex et al., 1995; Jiang et al., 2000; Chen et al., 2009). It should be noted, however, that other studies have suggested that the principle action of the sensory vagus in these antigen challenged models may be to monitor GI activity during the anaphylactic response, rather than playing a critical role in symptom generation (Scott et al., 1998). In this regard, studies have noted that the role of vagal afferents to inhibit nociceptive signaling may have temporally restricted actions, triggering endogenous antinociception at the early stages of allergen challenge and thereafter declining over time (Chen et al., 2009).

In part, this time-dependent decline in response may be related to the functional presence and activity of 5-HT_3_ receptors on vagal afferents; prolonged activation of 5-HT_3_ receptors leads to receptor desensitization and internalization (Freeman et al., 2006) and a decrease in receptor mRNA levels has been observed following chronic immune challenge (Chen et al., 2009). Also of relevance in this regard are the altered expression levels of serotonin transporters, particularly the serotonin-selective reuptake transporter (SERT) in several visceral hypersensitivity disorders. 5-HT signaling is terminated by reuptake into intestinal epithelium or nerve terminals via specialized transporter systems; alterations in SERT levels, therefore, are critical in regulating the availability, activity and duration of 5-HT signaling. SERT expression is downregulated in several hypersensitivity disorders including intestinal inflammatory conditions such as IBD as well as some, but not all, patients with diarrhea-predominant IBS (Coates et al., 2004; Camilleri et al., 2007; Foley et al., 2011). It is unclear whether such alterations in SERT contribute to dysregulated vagal afferent signaling in these groups, however. It is also unclear whether SERT expression levels are altered centrally in response to visceral hypersensitivity disorders; blocking SERT activity in the brainstem *decreases* glutamatergic synaptic transmission from the central terminals of vagal afferents due to the activation of presynaptic 5-HT_1A_ receptors, the activity of which are more tightly regulated by physical proximity to uptake transporters (Hosford et al., 2014). An increase in brainstem 5-HT levels in response to altered SERT activity may, therefore, have the potential to dramatically alter the gain of GI vagal afferent information transfer.

Many chronic pain syndromes, including IBS, are significantly more prevalent in women suggesting a role for gonadal hormones in the modulation of visceral sensitivity (Mulak et al., 2014). Estradiol has been shown to increase the secretion of 5-HT from intestinal mucosal mast cells in rats (Yan et al., 2014) causing the activation vagal afferent 5-HT_3_ receptors and an inhibition of the visceromotor response to colorectal distention in rats. It should also be noted, however, that estradiol has pronociceptive actions via spinal mechanisms; an imbalance between vagal antinociceptive and spinal pronociceptive pathways as estrogen levels fluctuate during the menstrual cycle may potentially exacerbate visceral sensitivity in susceptible IBS females (Yan et al., 2014).

Although, the regulation of food intake and energy homeostasis is generally considered to involve the integration of “higher” CNS centers with autonomic nuclei, the role of vago-vagal neurocircuits in the regulation of early satiety signaling has been the subject of renewed attention by several laboratory groups (Page et al., 2012; Dockray, 2013; de Lartigue, 2014; Kentish and Page, 2014). Diet-induced obesity is known to compromise the excitability and responsiveness of GI vagal afferent fibers (Covasa et al., 2000a,b; Swartz et al., 2010; Kentish et al., 2012) and neurons (Donovan et al., 2007; Paulino et al., 2009; Daly et al., 2011; de Lartigue et al., 2011). The mechanism responsible for this attenuated excitability has not been elucidated fully although studies in both obese mice and rats demonstrating a decreased membrane input resistance and increased membrane capacitance are suggestive of an increase in resting background potassium conductance(s) (Daly et al., 2011; Browning et al., 2013b). Studies have suggested that 5-HT_3_ receptor expression is downregulated following short term exposure to a high fat diet (Nefti et al., 2009) and 5-HT_3_-dependent activation of vagal afferent neurons is attenuated in diet-induced obese mice (Daly et al., 2011) but it is unclear whether this reflects the obesity-induced generalized decrease in vagal afferent excitability or a more specific decline in 5-HT_3_ function. In our recent studies in pre-obese rats fed a high fat diet, however, we have not observed an attenuated or compromised response of gastric vagal afferent fibers to 5-HT_3_ receptor activation (Troy et al., 2015), suggesting that obesity itself, rather than exposure to a high fat diet, may be responsible for the compromised 5-HT_3_ receptor signaling.

Evidence from several fields have suggested that vagal neurocircuits are not static relay networks where afferent activation triggers formulaic and unmodulated output responses. Rather, vagal neurocircuits display a remarkable degree of plasticity with their excitability and responsiveness being modulated readily by diet, insult or injury (Browning and Travagli, 2001, 2011; Bielefeldt et al., 2002a,b; Kollarik and Undem, 2002; Dang et al., 2004; Kang et al., 2004, 2005; Tolstykh et al., 2004; Hermes et al., 2008; Kentish et al., 2012, 2014; Browning et al., 2013b). In this regard, it is interesting to note that allergic challenge in sensitized animals induces a 5-HT_3_-dependent exposure of tachykinin receptor responses in respiratory vagal afferents and neurons (Weinreich et al., 1997; Moore et al., 1999, 2000, 2002); similar changes in GI afferents and neurons may also play a role in visceral hypersensitivity. Also of relevance is the finding that, despite being asynaptic, cross-talk exists between nodose ganglion neurons, where excitation of one neuron may influence that of a neighboring neuron by neurotransmitter-dependent and -independent means (Oh and Weinreich, 2002). The nodose ganglion (or nodose-jugular complex) houses the cell bodies of all vagal afferent neurons; although a generalized viscerotopic organization of soma has been proposed with neurons innervating the esophagus and aortic depressor nerve being located more rostrally with neurons innervating the stomach and pancreas being located more caudally (Zhuo et al., 1997), clearly cross-talk between neurons, may provide a means by which neurons innervating different visceral organs, or different GI areas, may influence or modulate activity of unrelated neurons.

## Conclusions

5-HT and 5-HT_3_ receptors in particular, are clearly important in gut-brain signaling and in the regulation and modulation of several vagally-mediated GI physiological reflexes and may play additional roles in several pathophysiological conditions. 5-HT_3_ receptors also appear open to modulation; extracellular glucose levels, for example, traffic 5-HT_3_ receptors to and from the neuronal membrane of GI nodose neurons amplifying or attenuating the 5-HT-induced response, while some, but not all, reports suggest alterations in receptor function by diet induced obesity. It would be surprising, however, if dietary micronutrients were the only mediators 5-HT_3_ receptor plasticity. Antigen challenge, for example, has been shown to induce 5-HT_3_ receptor dependent unmasking of tachykinin functions in respiratory nodose neurons; future studies investigating whether similar changes occur in GI nodose neurons may provide novel treatment strategies for allergen induced visceral hypersensitivity. Also intriguing is the apparent dichotomy between vagal afferent 5-HT_3_ responses; excessive activation of vagal afferent 5-HT_3_ receptors induces nausea and vomiting whereas several reports suggest an initial, temporally discrete anti-nociceptive response in stress-induced hypersensitivity. These (and other) diverse 5-HT_3_ receptor-dependent responses present obvious problems to the therapeutic use of receptor selective agonists or antagonists yet their more readily accessible nature means that vagal afferent 5-HT_3_ receptors still present an attract target for translational research.

### Conflict of interest statement

The author declares that the research was conducted in the absence of any commercial or financial relationships that could be construed as a potential conflict of interest.

## References

[B1] Andresen M. C. Kunze D. L. (1994). Nucleus tractus solitarius–gateway to neural circulatory control. Annu. Rev. Physiol. 56, 93–116. 10.1146/annurev.ph.56.030194.000521 7912060

[B2] Andrews P. L. Davis C. J. Bingham S. Davidson H. I. Hawthorn J. Maskell L. (1990). The abdominal visceral innervation and the emetic reflex: pathways, pharmacology, and plasticity. Can. J. Physiol. Pharmacol. 68, 325–345. 10.1139/y90-047 2178756

[B3] Andrews P. L. Horn C. C. (2006). Signals for nausea and emesis: implications for models of upper gastrointestinal diseases. Auton. Neurosci. 125, 100–115. 10.1016/j.autneu.2006.01.008 16556512 PMC2658708

[B4] Babic T. Browning K. N. (2014). The role of vagal neurocircuits in the regulation of nausea and vomiting. Eur. J. Pharmacol. 722, 38–47. 10.1016/j.ejphar.2013.08.047 24184670 PMC3893663

[B5] Babic T. Troy A. E. Fortna S. R. Browning K. N. (2012). Glucose-dependent trafficking of 5-HT(3) receptors in rat gastrointestinal vagal afferent neurons. Neurogastroenterol. Motil. 24, e476–e488. 10.1111/j.1365-2982.2012.01987.x 22845622 PMC3440531

[B6] Baptista V. Browning K. N. Travagli R. A. (2007). Effects of cholecystokinin-8s in the nucleus tractus solitarius of vagally deafferented rats. Am. J. Physiol. Regul. Integr. Comp. Physiol. 292, R1092–R1100. 10.1152/ajpregu.00517.2006 17122331 PMC3062489

[B7] Berthoud H.-R. Carlson N. R. Powley T. L. (1991). Topography of efferent vagal innervation of the rat gastrointestinal tract. Am. J. Physiol. 260, R200–R207. 1992820 10.1152/ajpregu.1991.260.1.R200

[B8] Bertrand P. P. Kunze W. A. Furness J. B. Bornstein J. C. (2000). The terminals of myenteric intrinsic primary afferent neurons of the guinea-pig ileum are excited by 5-hydroxytryptamine acting at 5- hydroxytryptamine-3 receptors. Neuroscience 101, 459–469. 10.1016/S0306-4522(00)00363-8 11074168

[B9] Beyak M. J. Grundy D. (2005). Vagal afferents innervating the gastrointestinal tract, in Advances in Vagal Afferent Neurobiology, eds Undem B. J. Weinreich D. (Boca Raton, FL: CRC Press), 315–350.

[B10] Bielefeldt K. Ozaki N. Gebhart G. F. (2002a). Experimental ulcers alter voltage-sensitive sodium currents in rat gastric sensory neurons. Gastroenterology 122, 394–405. 10.1053/gast.2002.31026 11832454

[B11] Bielefeldt K. Ozaki N. Gebhart G. F. (2002b). Mild gastritis alters voltage-sensitive sodium currents in gastric sensory neurons in rats. Gastroenterology 122, 752–761. 10.1053/gast.2002.31901 11875008

[B12] Blackshaw L. A. Grundy D. (1993). Effects of 5-hydroxytryptamine (5-HT) on the discharge of vagal mechanoreceptors and motility in the upper gastrointestinal tract of the ferret. J. Auton. Nerv. Syst. 45, 51–59. 10.1016/0165-1838(93)90361-W 8227964

[B13] Blakelock R. T. Beasley S. W. (2003). Infection and the gut. Semin. Pediatr. Surg. 12, 265–274. 10.1053/j.sempedsurg.2003.08.008 14655166

[B14] Bradesi S. Lao L. McLean P. G. Winchester W. J. Lee K. Hicks G. A.. (2007). Dual role of 5-HT_3_ receptors in a rat model of delayed stress-induced visceral hyperalgesia. Pain 130, 56–65. 10.1016/j.pain.2006.10.028 17161536

[B15] Browning K. N. (2013). Modulation of gastrointestinal vagal neurocircuits by hyperglycemia. Front. Neurosci. 7:217. 10.3389/fnins.2013.00217 24324393 PMC3840437

[B16] Browning K. N. Babic T. Holmes G. M. Swartz E. M. Travagli R. A. (2013a). A critical re-evaluation of the specificity of action of perivagal capsaicin. J. Physiol. 591, 1563–1580. 10.1113/jphysiol.2012.246827 23297311 PMC3607173

[B17] Browning K. N. Fortna S. R. Hajnal A. (2013b). Roux-en-Y gastric bypass reverses the effects of diet-induced obesity to inhibit the responsiveness of central vagal motoneurones. J. Physiol. 591, 2357–2372. 10.1113/jphysiol.2012.249268 23459752 PMC3650700

[B18] Browning K. N. Travagli R. A. (2001). The peptide TRH uncovers the presence of presynaptic 5-HT1A receptors via activation of a second messenger pathway in the rat dorsal vagal complex. J. Physiol. 531, 425–435. 10.1111/j.1469-7793.2001.0425i.x 11230515 PMC2278482

[B19] Browning K. N. Travagli R. A. (2011). Plasticity of vagal brainstem circuits in the control of gastrointestinal function. Auton. Neurosci. 161, 6–13. 10.1016/j.autneu.2010.11.001 21147043 PMC3061976

[B20] Browning K. N. Travagli R. A. (2014). Central nervous system control of gastrointestinal motility and secretion and modulation of gastrointestinal functions. Compr. Physiol. 4, 1339–1368. 10.1002/cphy.c130055 25428846 PMC4858318

[B21] Bulbring E. Lin R. C. (1958). The effect of intraluminal application of 5-hydroxytryptamine and 5-hydroxytryptophan on peristalsis; the local production of 5-HT and its release in relation to intraluminal pressure and propulsive activity. J. Physiol. 140, 381–407. 13514713 PMC1358765

[B22] Callera J. C. Sevoz C. Laguzzi R. Machado B. H. (1997). Microinjection of a serotonin3 receptor agonist into the NTS of unanesthetized rats inhibits the bradycardia evoked by activation of the baro- and chemoreflexes. J. Auton. Nerv. Syst. 63, 127–136. 10.1016/S0165-1838(96)00140-3 9138244

[B23] Camilleri M. Andrews C. N. Bharucha A. E. Carlson P. J. Ferber I. Stephens D.. (2007). Alterations in expression of p11 and SERT in mucosal biopsy specimens of patients with irritable bowel syndrome. Gastroenterology 132, 17–25. 10.1053/j.gastro.2006.11.020 17241856 PMC2474784

[B24] Castex N. Fioramonti J. Fargeas M. J. Bueno L. (1995). c-fos expression in specific rat brain nuclei after intestinal anaphylaxis: involvement of 5-HT_3_ receptors and vagal afferent fibers. Brain Res. 688, 149–160. 10.1016/0006-8993(95)00526-V 8542301

[B25] Chen S. Li J. Zhang L. Dong X. Gao W. Mo J.. (2009). 5-HT 3 receptors mediate the time-dependent vagal afferent modulation of nociception during chronic food allergen-sensitized visceral hyperalgesia in rats. Neurogastroenterol. Motil. 21, 1222–e113. 10.1111/j.1365-2982.2009.01335.x 19558425

[B26] Chen S. L. Wu X. Y. Cao Z. J. Fan J. Wang M. Owyang C.. (2008). Subdiaphragmatic vagal afferent nerves modulate visceral pain. Am. J. Physiol. Gastrointest. Liver Physiol. 294, G1441–G1449. 10.1152/ajpgi.00588.2007 18420825 PMC3222235

[B27] Coates M. D. Mahoney C. R. Linden D. R. Sampson J. E. Chen J. Blaszyk H.. (2004). Molecular defects in mucosal serotonin content and decreased serotonin reuptake transporter in ulcerative colitis and irritable bowel syndrome. Gastroenterology 126, 1657–1664. 10.1053/j.gastro.2004.03.013 15188158

[B28] Coleman N. S. Marciani L. Blackshaw E. Wright J. Parker M. Yano T.. (2003). Effect of a novel 5-HT_3_ receptor agonist MKC-733 on upper gastrointestinal motility in humans. Aliment. Pharmacol. Ther. 18, 1039–1048. 10.1046/j.1365-2036.2003.01797.x 14616171

[B29] Cottrell G. T. Ferguson A. V. (2004). Sensory circumventricular organs: central roles in integrated autonomic regulation. Regul. Pept. 117, 11–23. 10.1016/j.regpep.2003.09.004 14687696

[B30] Covasa M. Grahn J. Ritter R. C. (2000a). High fat maintenance diet attenuates hindbrain neuronal response to CCK. Regul. Pept. 86, 83–88. 10.1016/S0167-0115(99)00084-1 10672906

[B31] Covasa M. Grahn J. Ritter R. C. (2000b). Reduced hindbrain and enteric neuronal response to intestinal oleate in rats maintained on high-fat diet. Auton. Neurosci. 84, 8–18. 10.1016/S1566-0702(00)00176-4 11109985

[B32] Cui R. J. Roberts B. L. Zhao H. Zhu M. Appleyard S. M. (2012). Serotonin activates catecholamine neurons in the solitary tract nucleus by increasing spontaneous glutamate inputs. J. Neurosci. 32, 16530–16538. 10.1523/JNEUROSCI.1372-12.2012 23152635 PMC3752146

[B33] Daly D. M. Park S. J. Valinsky W. C. Beyak M. J. (2011). Impaired intestinal afferent nerve satiety signalling and vagal afferent excitability in diet induced obesity in the mouse. J. Physiol. 589, 2857–2870. 10.1113/jphysiol.2010.204594 21486762 PMC3112560

[B34] Dang K. Bielefeldt K. Gebhart G. F. (2004). Gastric ulcers reduce A-type potassium currents in rat gastric sensory ganglion neurons. Am. J. Physiol. Gastrointest. Liver Physiol. 286, G573–G579. 10.1152/ajpgi.00258.2003 14525728

[B35] Darmani N. A. Johnson J. C. (2004). Central and peripheral mechanisms contribute to the antiemetic actions of delta-9-tetrahydrocannabinol against 5-hydroxytryptophan-induced emesis. Eur. J. Pharmacol. 488, 201–212. 10.1016/j.ejphar.2004.02.018 15044052

[B36] Darmani N. A. Ray A. P. (2009). Evidence for a re-evaluation of the neurochemical and anatomical bases of chemotherapy-induced vomiting. Chem. Rev. 109, 3158–3199. 10.1021/cr900117p 19522506

[B37] de Lartigue G. (2014). Putative roles of neuropeptides in vagal afferent signaling. Physiol. Behav. 136, 155–169. 10.1016/j.physbeh.2014.03.011 24650553 PMC4167981

[B38] de Lartigue G. de La Serre C. B. Raybould H. E. (2011). Vagal afferent neurons in high fat diet-induced obesity; intestinal microflora, gut inflammation and cholecystokinin. Physiol. Behav. 105, 100–105. 10.1016/j.physbeh.2011.02.040 21376066 PMC3156356

[B39] Derkach V. Surprenant A. North R. A. (1989). 5-HT_3_ receptors are membrane ion channels. Nature 339, 706–709. 10.1038/339706a0 2472553

[B40] Dockray G. J. (2013). Enteroendocrine cell signalling via the vagus nerve. Curr. Opin. Pharmacol. 13, 954–958. 10.1016/j.coph.2013.09.007 24064396

[B41] Donovan M. J. Paulino G. Raybould H. E. (2007). CCK(1) receptor is essential for normal meal patterning in mice fed high fat diet. Physiol. Behav. 92, 969–974. 10.1016/j.physbeh.2007.07.003 18023701 PMC2675541

[B42] Dunn-Meynell A. A. Sanders N. M. Compton D. Becker T. C. Eiki J. Zhang B. B.. (2009). Relationship among brain and blood glucose levels and spontaneous and glucoprivic feeding. J. Neurosci. 29, 7015–7022. 10.1523/JNEUROSCI.0334-09.2009 19474328 PMC2728115

[B43] Endo T. Minami M. Hirafuji M. Ogawa T. Akita K. Nemoto M.. (2000). Neurochemistry and neuropharmacology of emesis - the role of serotonin. Toxicology 153, 189–201. 10.1016/S0300-483X(00)00314-0 11090957

[B44] Endo T. Minami M. Monma Y. Saito H. Takeuchi M. (1990). Emesis-related biochemical and histopathological changes induced by cisplatin in the ferret. J. Toxicol. Sci. 15, 235–244. 10.2131/jts.15.235 1707101

[B45] Foley S. Garsed K. Singh G. Duroudier N. P. Swan C. Hall I. P.. (2011). Impaired uptake of serotonin by platelets from patients with irritable bowel syndrome correlates with duodenal immune activation. Gastroenterology 140, 1434–1443. 10.1053/j.gastro.2011.01.052 21315720

[B46] Freeman S. L. Glatzle J. Robin C. S. Valdellon M. Sternini C. Sharp J. W.. (2006). Ligand-induced 5-HT_3_ receptor internalization in enteric neurons in rat ileum. Gastroenterology 131, 97–107. 10.1053/j.gastro.2006.04.013 16831594

[B47] Fry M. Ferguson A. V. (2007). The sensory circumventricular organs: brain targets for circulating signals controlling ingestive behavior. Physiol. Behav. 91, 413–423. 10.1016/j.physbeh.2007.04.003 17531276

[B48] Gaudin-Chazal G. Portalier P. Barrit M. C. Puizillout J. J. (1982). Serotonin-like immunoreactivity in paraffin-sections of the nodose ganglia of the cat. Neurosci. Lett. 33, 169–172. 10.1016/0304-3940(82)90246-4 6759990

[B49] Gentilcore D. Little T. J. Feinle-Bisset C. Samsom M. Smout A. J. Horowitz M.. (2007). Role of 5-hydroxytryptamine mechanisms in mediating the effects of small intestinal glucose on blood pressure and antropyloroduodenal motility in older subjects. Am. J. Physiol. Gastrointest. Liver Physiol. 293, G692–G698. 10.1152/ajpgi.00199.2007 17656445

[B50] Gershon M. D. Tack J. (2007). The serotonin signaling system: from basic understanding to drug development for functional GI disorders. Gastroenterology 132, 397–414. 10.1053/j.gastro.2006.11.002 17241888

[B51] Glatzle J. Sternini C. Robin C. Zittel T. T. Wong H. Reeve J. R. Jr.. (2002). Expression of 5-HT_3_ receptors in the rat gastrointestinal tract. Gastroenterology 123, 217–226. 10.1053/gast.2002.34245 12105850

[B52] Glaum S. R. Brooks P. A. Spyer K. M. Miller R. J. (1992). 5-Hydroxytryptamine-3 receptors modulate synaptic activity in the rat nucleus tractus solitarius *in vitro*. Brain Res. 589, 62–68. 10.1016/0006-8993(92)91162-8 1422823

[B53] Grabauskas G. Song I. Zhou S. Y. Owyang C. (2010). Electrophysiological identifications of glucose-sensing neurons in the rat nodose ganglia. J. Physiol. 588, 617–632. 10.1113/jphysiol.2009.182147 20008464 PMC2828136

[B54] Gwynne R. M. Bornstein J. C. (2007). Local inhibitory reflexes excited by mucosal application of nutrient amino acids in guinea pig jejunum. Am. J. Physiol. Gastrointest. Liver Physiol. 292, G1660–G1670. 10.1152/ajpgi.00580.2006 17347449

[B55] Hagbom M. Istrate C. Engblom D. Karlsson T. Rodriguez-Diaz J. Buesa J.. (2011). Rotavirus stimulates release of serotonin (5-HT) from human enterochromaffin cells and activates brain structures involved in nausea and vomiting. PLoS Pathog. 7:e1002115. 10.1371/journal.ppat.1002115 21779163 PMC3136449

[B56] Hawthorn J. Ostler K. J. Andrews P. L. (1988). The role of the abdominal visceral innervation and 5-hydroxytryptamine M-receptors in vomiting induced by the cytotoxic drugs cyclophosphamide and cis-platin in the ferret. Q. J. Exp. Physiol. 73, 7–21. 10.1113/expphysiol.1988.sp003124 3347698

[B57] Hayes M. R. Covasa M. (2006a). Dorsal hindbrain 5-HT_3_ receptors participate in control of meal size and mediate CCK-induced satiation. Brain Res. 1103, 99–107. 10.1016/j.brainres.2006.05.058 16793030

[B58] Hayes M. R. Covasa M. (2006b). Gastric distension enhances CCK-induced Fos-like immunoreactivity in the dorsal hindbrain by activating 5-HT_3_ receptors. Brain Res. 1088, 120–130. 10.1016/j.brainres.2006.03.018 16630589

[B59] Hermes S. M. Mitchell J. L. Silverman M. B. Lynch P. J. McKee B. L. Bailey T. W.. (2008). Sustained hypertension increases the density of AMPA receptor subunit, GluR1, in baroreceptive regions of the nucleus tractus solitarii of the rat. Brain Res. 1187, 125–136. 10.1016/j.brainres.2007.10.041 18031714 PMC2225988

[B60] Hillsley K. Grundy D. (1998). Sensitivity to 5-hydroxytryptamine in different afferent subpopulations within mesenteric nerves supplying the rat jejunum. J. Physiol. 509(Pt 3), 717–727. 10.1111/j.1469-7793.1998.717bm.x 9596794 PMC2230991

[B61] Hillsley K. Kirkup A. J. Grundy D. (1998). Direct and indirect actions of 5-hydroxytryptamine on the discharge of mesenteric afferent fibres innervating the rat jejunum. J. Physiol. 506(Pt 2), 551–561. 10.1111/j.1469-7793.1998.551bw.x 9490878 PMC2230728

[B62] Horn C. C. Richardson E. J. Andrews P. L. Friedman M. I. (2004). Differential effects on gastrointestinal and hepatic vagal afferent fibers in the rat by the anti-cancer agent cisplatin. Auton. Neurosci. 115, 74–81. 10.1016/j.autneu.2004.08.011 15507408

[B63] Hosford P. S. Mifflin S. W. Ramage A. G. (2014). 5-hydroxytryptamine-mediated neurotransmission modulates spontaneous and vagal-evoked glutamate release in the nucleus of the solitary tract effect of uptake blockade. J. Pharmacol. Exp. Ther. 349, 288–296. 10.1124/jpet.113.211334 24618127

[B64] Houghton L. A. Atkinson W. Whitaker R. P. Whorwell P. J. Rimmer M. J. (2003). Increased platelet depleted plasma 5-hydroxytryptamine concentration following meal ingestion in symptomatic female subjects with diarrhoea predominant irritable bowel syndrome. Gut 52, 663–670. 10.1136/gut.52.5.663 12692050 PMC1773651

[B65] Huang J. Spier A. D. Pickel V. M. (2004). 5-HT_3_A receptor subunits in the rat medial nucleus of the solitary tract: subcellular distribution and relation to the serotonin transporter. Brain Res. 1028, 156–169. 10.1016/j.brainres.2004.09.009 15527741

[B66] Jeggo R. D. Kellett D. O. Wang Y. Ramage A. G. Jordan D. (2005). The role of central 5-HT_3_ receptors in vagal reflex inputs to neurones in the nucleus tractus solitarius of anaesthetized rats. J. Physiol. 566, 939–953. 10.1113/jphysiol.2005.085845 15905216 PMC1464782

[B67] Jensen G. M. Grondahl M. L. Nielsen C. G. Skadhauge E. Olsen J. E. Hansen M. B. (1997). Effect of ondansetron on Salmonella typhimurium-induced net fluid accumulation in the pig jejunum *in vivo*. Comp. Biochem. Physiol. A Physiol. 118, 297–299. 10.1016/S0300-9629(96)00308-8 9366059

[B68] Jiang W. Kreis M. E. Eastwood C. Kirkup A. J. Humphrey P. P. Grundy D. (2000). 5-HT(3) and histamine H(1) receptors mediate afferent nerve sensitivity to intestinal anaphylaxis in rats. Gastroenterology 119, 1267–1275. 10.1053/gast.2000.19461 11054384

[B69] Jordan D. (2005). Vagal control of the heart: central serotonergic (5-HT) mechanisms. Exp. Physiol. 90, 175–181. 10.1113/expphysiol.2004.029058 15604109

[B70] Kang Y. M. Bielefeldt K. Gebhart G. F. (2004). Sensitization of mechanosensitive gastric vagal afferent fibers in the rat by thermal and chemical stimuli and gastric ulcers. J. Neurophysiol. 91, 1981–1989. 10.1152/jn.01097.2003 15069095

[B71] Kang Y. M. Lamb K. Gebhart G. F. Bielefeldt K. (2005). Experimentally induced ulcers and gastric sensory-motor function in rats. Am. J. Physiol. Gastrointest. Liver Physiol. 288, G284–G291. 10.1152/ajpgi.00250.2004 15388487

[B72] Keating D. J. Spencer N. J. (2010). Release of 5-hydroxytryptamine from the mucosa is not required for the generation or propagation of colonic migrating motor complexes. Gastroenterology 138, 659–670. 10.1053/j.gastro.2009.09.020 19782081

[B73] Kentish S. J. O'donnell T. A. Wittert G. A. Page A. J. (2014). Diet-dependent modulation of gastro-oesphageal vagal afferent mechanosensitivity by endogenous nitric oxide. J. Physiol. 592, 3287–3301. 10.1113/jphysiol.2014.272674 24879868 PMC4146376

[B74] Kentish S. J. Page A. J. (2014). Plasticity of gastro-intestinal vagal afferent endings. Physiol. Behav. 136, 170–178. 10.1016/j.physbeh.2014.03.012 24657740

[B75] Kentish S. Li H. Philp L. K. O'donnell T. A. Isaacs N. J. Young R. L.. (2012). Diet-induced adaptation of vagal afferent function. J. Physiol. 590, 209–221. 10.1113/jphysiol.2011.222158 22063628 PMC3300057

[B76] Kollarik M. Undem B. J. (2002). Mechanisms of acid-induced activation of airway afferent nerve fibres in guinea-pig. J. Physiol. 543, 591–600. 10.1113/jphysiol.2002.022848 12205192 PMC2290522

[B77] Kreis M. E. Jiang W. Kirkup A. J. Grundy D. (2002). Cosensitivity of vagal mucosal afferents to histamine and 5-HT in the rat jejunum. Am. J. Physiol. Gastrointest. Liver Physiol. 283, G612–G617. 10.1152/ajpgi.00206.2001 12181174

[B78] Lacolley P. Owen J. R. Sandock K. Lewis T. H. Bates J. N. Robertson T. P.. (2006a). 5-HT activates vagal afferent cell bodies *in vivo*: role of 5-HT2 and 5-HT_3_ receptors. Neuroscience 143, 273–287. 10.1016/j.neuroscience.2006.07.032 17029799

[B79] Lacolley P. Owen J. R. Sandock K. Lewis T. H. Bates J. N. Robertson T. P.. (2006b). Occipital artery injections of 5-HT may directly activate the cell bodies of vagal and glossopharyngeal afferent cell bodies in the rat. Neuroscience 143, 289–308. 10.1016/j.neuroscience.2006.08.047 17029801

[B80] Leal-Cardoso H. Koschorke G. M. Taylor G. E. Weinreich D. (1993). Electrophysiological properties and chemosensitivity of acutely isolated nodose ganglion neurons of the rabbit. JANS 45, 29–39. 10.1016/0165-1838(93)90359-3 7901264

[B81] Leslie R. A. Reynolds D. J. M. Andrews P. L. Grahame-Smith D. G. Davis C. J. Harvey J. M. (1990). Evidence for presynaptic 5-hydroxytryptamine _3_ recognition sites on vagal afferent terminals in the brainstem of the ferret. Neuroscience 38, 667–673. 10.1016/0306-4522(90)90060-H 2176720

[B82] Liu Y. Hamaue N. Endo T. Hirafuji M. Minami M. (2003). 5-hydroxytryptamine (5-HT) concentrations in the hippocampus, the hypothalamus and the medulla oblongata related to cisplatin-induced pica of rats. Res. Commun. Mol. Pathol. Pharmacol. 113-114, 97–113. 15686111

[B83] MacGregor I. L. Gueller R. Watts H. D. Meyer J. H. (1976). The effect of acute hyperglycemia on gastric emptying in man. Gastroenterology 70, 190–196. 765178

[B84] Mawe G. M. Hoffman J. M. (2013). Serotonin signalling in the gut–functions, dysfunctions and therapeutic targets. Nat. Rev. Gastroenterol. Hepatol. 10, 473–486. 10.1038/nrgastro.2013.105 23797870 PMC4048923

[B85] Mazda T. Yamamoto H. Fujimura M. Fujimiya M. (2004). Gastric distension-induced release of 5-HT stimulates c-fos expression in specific brain nuclei via 5-HT_3_ receptors in conscious rats. Am. J. Physiol. Gastrointest. Liver Physiol. 287, G228–G235. 10.1152/ajpgi.00373.2003 14684379

[B86] Mei N. (1978). Vagal glucoreceptors in the small intestine of the cat. J. Physiol. 282, 485–506. 10.1113/jphysiol.1978.sp012477 722554 PMC1282753

[B87] Meller S. T. Lewis S. J. Brody M. J. Gebhart G. F. (1992). Vagal afferent-mediated inhibition of a nociceptive reflex by i.v. serotonin in the rat. II. Role of 5-HT receptor subtypes. Brain Res. 585, 71–86. 10.1016/0006-8993(92)91192-H 1511336

[B88] Moore K. A. Oh E. J. Weinreich D. (2002). 5-HT(3) receptors mediate inflammation-induced unmasking of functional tachykinin responses *in vitro*. J. Appl. Physiol. 92, 2529–2534. 10.1152/japplphysiol.00974.2001 12015369

[B89] Moore K. A. Taylor G. E. Weinreich D. (1999). Serotonin unmasks functional NK-2 receptors in vagal sensory neurons of the guinea-pig. J. Physiol. 514, 111–124. 10.1111/j.1469-7793.1999.111af.x 9831720 PMC2269056

[B90] Moore K. A. Undem B. J. Weinreich D. (2000). Antigen inhalation unmasks NK-2 tachykinin receptor-mediated responses in vagal afferents. Am. J. Resp. Crit. Care Med. 161, 232–236. 10.1164/ajrccm.161.1.9903091 10619825

[B91] Mulak A. Taché Y. Larauche M. (2014). Sex hormones in the modulation of irritable bowel syndrome. World J. Gastroenterol. 20, 2433–2448. 10.3748/wjg.v20.i10.2433 24627581 PMC3949254

[B92] Mussa B. M. Sartor D. M. Verberne A. J. (2008). Activation of cholecystokinin (CCK 1) and serotonin (5-HT 3) receptors increases the discharge of pancreatic vagal afferents. Eur. J. Pharmacol. 601, 198–206. 10.1016/j.ejphar.2008.11.007 19026634

[B93] Mussa B. M. Sartor D. M. Verberne A. J. (2010). Dorsal vagal preganglionic neurons: differential responses to CCK1 and 5-HT_3_ receptor stimulation. Auton. Neurosci. 156, 36–43. 10.1016/j.autneu.2010.03.001 20346737

[B94] Nakata-Fukuda M. Hirata T. Keto Y. Yamano M. Yokoyama T. Uchiyama Y. (2014). Inhibitory effect of the selective serotonin 5-HT(3) receptor antagonist ramosetron on duodenal acidification-induced gastric hypersensitivity in rats. Eur. J. Pharmacol. 731, 88–92. 10.1016/j.ejphar.2014.02.040 24632457

[B95] Nefti W. Chaumontet C. Fromentin G. Tomé D. Darcel N. (2009). A high-fat diet attenuates the central response to within-meal satiation signals and modifies the receptor expression of vagal afferents in mice. Am. J. Physiol. Regul. Integr. Comp. Physiol. 296, R1681–R1686. 10.1152/ajpregu.90733.2008 19297544

[B96] Nosjean A. Compoint C. Buisseret-Delmas C. Orer H. S. Merahi N. Puizillout J. J.. (1990). Serotonergic projections from the nodose ganglia to the nucleus tractus solitarius: an immunohistochemical and double labeling study in the rat. Neurosci. Lett. 114, 22–26. 10.1016/0304-3940(90)90422-6 1696365

[B97] Oh E. J. Weinreich D. (2002). Chemical communication between vagal afferent somata in nodose Ganglia of the rat and the Guinea pig *in vitro*. J. Neurophysiol. 87, 2801–2807. 12037182 10.1152/jn.2002.87.6.2801

[B98] Page A. J. Symonds E. Peiris M. Blackshaw L. A. Young R. L. (2012). Peripheral neural targets in obesity. Br. J. Pharmacol. 166, 1537–1558. 10.1111/j.1476-5381.2012.01951.x 22432806 PMC3419899

[B99] Paintal A. S. (1951). A method of locating the receptors of visceral afferent fibers. J. Physiol. 124, 166–172. 10.1113/jphysiol.1954.sp005095 13163877 PMC1366250

[B100] Paulino G. Barbier de la Serre C. Knotts T. A. Oort P. J. Newman J. W. Adams S. H.. (2009). Increased expression of receptors for orexigenic factors in nodose ganglion of diet-induced obese rats. Am. J. Physiol. Endocrinol. Metab. 296, E898–E903. 10.1152/ajpendo.90796.2008 19190260 PMC2670626

[B101] Peters J. A. Malone H. M. Lambert J. J. (1993). An electrophysiological investigation of the properties of 5-HT_3_ receptors of rabbit nodose ganglion neurones in culture. Br. J. Pharmacol. 110, 665–676. 10.1111/j.1476-5381.1993.tb13863.x 7694755 PMC2175932

[B102] Pires J. G. Silva S. R. Ramage A. G. Futuro-Neto H. A. (1998). Evidence that 5-HT_3_ receptors in the nucleus tractus solitarius and other brainstem areas modulate the vagal bradycardia evoked by activation of the von Bezold-Jarisch reflex in the anesthetized rat. Brain Res. 791, 229–234. 10.1016/S0006-8993(98)00109-7 9593908

[B103] Powley T. L. Phillips R. J. (2002). Musings on the wanderer: what's new in our understanding of vago-vagal reflexes? I. Morphology and topography of vagal afferents innervating the GI tract. Am. J. Physiol. Gastrointest. Liver Physiol. 283, G1217–G1225. 10.1152/ajpgi.00249.2002 12388183

[B104] Powley T. L. Spaulding R. A. Haglof S. A. (2011). Vagal afferent innervation of the proximal gastrointestinal tract mucosa: chemoreceptor and mechanoreceptor architecture. J. Comp. Neurol. 519, 644–660. 10.1002/cne.22541 21246548 PMC3902787

[B105] Ramage A. G. Mifflin S. W. (1998). Vagal-evoked excitation of a sub-population of neurons int he nucleus of the solitary tract (NTS) involves 5-HT_3_ receptors in the anesthetized rat. J. Physiol. 509P, 129P.

[B106] Ramage A. G. Villalon C. M. (2008). 5-Hydroxytryptamine and cardiovascular regulation. Trends Pharmacol. Sci. 29, 472–481. 10.1016/j.tips.2008.06.009 19086344

[B107] Randich A. Gebhart D. F. (1992). Vagal afferent modulation of nociception. Brain Res. Rev. 17, 77–99. 10.1016/0165-0173(92)90009-B 1327371

[B108] Raybould H. E. Glatzle J. Robin C. Meyer J. H. Phan T. Wong H.. (2003). Expression of 5-HT_3_ receptors by extrinsic duodenal afferents contribute to intestinal inhibition of gastric emptying. Am. J. Physiol. Gastrointest. Liver Physiol. 284, G367–G372. 10.1152/ajpgi.00292.2001 12409280

[B109] Raybould H. E. Zittel T. T. (1995). Inhibition of gastric motility induced by intestinal glucose in awake rats: role of Na(+)-glucose co-transporter. Neurogastroenterol. Motil. 7, 9–14. 10.1111/j.1365-2982.1995.tb00203.x 7627868

[B110] Rayner C. K. Samsom M. Jones K. L. Horowitz M. (2001). Relationships of upper gastrointestinal motor and sensory function with glycemic control. Diabetes Care 24, 371–381. 10.2337/diacare.24.2.371 11213895

[B111] Ren K. Randich A. Gebhart G. F. (1990). Modulation of spinal nociceptive transmission from nuclei tractus solitarii: a relay for effects of vagal afferent stimulation. J. Neurophysiol. 63, 971–986. 1972739 10.1152/jn.1990.63.5.971

[B112] Reynolds D. J. Barber N. A. Grahame-Smith D. G. Leslie R. A. (1991). Cisplatin-evoked induction of c-fos protein in the brainstem of the ferret: the effect of cervical vagotomy and the anti-emetic 5-HT_3_ receptor antagonist granisetron (BRL 43694). Brain Res. 565, 231–236. 10.1016/0006-8993(91)91654-J 1668810

[B113] Scott R. B. Tan D. T. Miampamba M. Sharkey K. A. (1998). Anaphylaxis-induced alterations in intestinal motility: role of extrinsic neural pathways. Am. J. Physiol. 275, G812–G821. 9756513 10.1152/ajpgi.1998.275.4.G812

[B114] Sévoz C. Callera J. C. Machado B. H. Hamon M. Laguzzi R. (1997). Role of serotonin3 receptors in the nucleus tractus solitarii on the carotid chemoreflex. Am. J. Physiol. 272, H1250–H1259. 9087599 10.1152/ajpheart.1997.272.3.H1250

[B115] Sévoz C. Nosjean A. Callera J. C. Machado B. Hamon M. Laguzzi R. (1996). Stimulation of 5-HT_3_ receptors in the NTS inhibits the cardiac Bezold-Jarisch reflex response. Am. J. Physiol. 271, H80–H87. 8760161 10.1152/ajpheart.1996.271.1.H80

[B116] Spencer N. J. Nicholas S. J. Robinson L. Kyloh M. Flack N. Brookes S. J.. (2011). Mechanisms underlying distension-evoked peristalsis in guinea pig distal colon: is there a role for enterochromaffin cells? Am. J. Physiol. Gastrointest. Liver Physiol. 301, G519–G527. 10.1152/ajpgi.00101.2011 21700904

[B117] Steinbusch H. W. (1981). Distribution of serotonin-immunoreactivity in the central nervous system of the rat-cell bodies and terminals. Neuroscience 6, 557–618. 10.1016/0306-4522(81)90146-9 7017455

[B118] Swartz T. D. Savastano D. M. Covasa M. (2010). Reduced sensitivity to cholecystokinin in male rats fed a high-fat diet is reversible. J. Nutr. 140, 1698–1703. 10.3945/jn.110.124149 20592106

[B119] Takenaka R. Ohi Y. Haji A. (2011). Distinct modulatory effects of 5-HT on excitatory synaptic transmissions in the nucleus tractus solitarius of the rat. Eur. J. Pharmacol. 671, 45–52. 10.1016/j.ejphar.2011.09.164 21968141

[B120] Thor K. B. Helke C. J. (1987). Serotonin- and substance P-containing projections to the nucleus tractus solitarii of the rat. J. Comp. Neurol. 265, 275–293. 10.1002/cne.902650210 2447131

[B121] Thor K. B. Helke C. J. (1989). Serotonin and substance P colocalization in medullary projections to the nucleus tractus solitarius: dual-colour immunohistochemistry combined with retrograde tracing. J. Chem. Neuroanat. 2, 139–148. 2477037

[B122] Thor K. B. Hill K. M. Harrod C. Helke C. J. (1988). Immunohistochemical and biochemical analysis of serotonin and substance P colocalization in the nucleus tractus solitarii and associated afferent ganglia of the rat. Synapse 2, 225–231. 10.1002/syn.890020309 2463690

[B123] Tolstykh G. Belugin S. Mifflin S. (2004). Responses to GABA(A) receptor activation are altered in NTS neurons isolated from chronic hypoxic rats. Brain Res. 1006, 107–113. 10.1016/j.brainres.2004.01.060 15047029

[B124] Tonini M. (2005). 5-Hydroxytryptamine effects in the gut: the 3, 4, and 7 receptors. Neurogastroenterol. Motil. 17, 637–642. 10.1111/j.1365-2982.2005.00716.x 16185301

[B125] Travagli R. A. Hermann G. E. Browning K. N. Rogers R. C. (2006). Brainstem circuits regulating gastric function. Annu. Rev. Physiol. 68, 279–305. 10.1146/annurev.physiol.68.040504.094635 16460274 PMC3062484

[B126] Troy A. E. Simmonds S. S. Stocker S. D. Browning K. N. (2015). High fat diet attenuates glucose-dependent facilitation of 5-HT3 mediated responses in rat gastric vagal afferents. J. Physiol. [Epub ahead of print]. 10.1113/JP271558 26456775 PMC4704508

[B127] Tuladhar B. R. Kaisar M. Naylor R. J. (1997). Evidence for a 5-HT_3_ receptor involvement in the facilitation of peristalsis on mucosal application of 5-HT in the guinea pig isolated ileum. Br. J. Pharmacol. 122, 1174–1178. 10.1038/sj.bjp.0701503 9401783 PMC1565058

[B128] Vanuytsel T. Karamanolis G. Van O. L. Vos R. Tack J. (2011). Influence of ondansetron on gastric sensorimotor responses to short duodenal acid infusion in healthy volunteers. Neurogastroenterol. Motil. 23, 226–232, e115. 10.1111/j.1365-2982.2010.01631.x 21114584

[B129] Wan S. Browning K. N. (2008a). D-Glucose modulates synaptic transmission from the central terminals of vagal afferent fibers. Am. J. Physiol. Gastrointest. Liver Physiol. 294, G757–G763. 10.1152/ajpgi.00576.2007 18202107

[B130] Wan S. Browning K. N. (2008b). Glucose increases synaptic transmission from vagal afferent central nerve terminals via modulation of 5HT3 receptors. Am. J. Physiol. Gastrointest. Liver Physiol. 295, G1050–G1057. 10.1152/ajpgi.90288.2008 18801915 PMC6842884

[B131] Wang Y. Ramage A. G. Jordan D. (1996). Mediation by 5-HT_3_ receptors of an excitatory effect of 5-HT on dorsal vagal preganglionic neurones in anesthetized rats: an ionophoretic study. Br. J. Pharmacol. 118, 1697–1704. 10.1111/j.1476-5381.1996.tb15594.x 8842434 PMC1909830

[B132] Weinreich D. Moore K. A. Taylor G. E. (1997). Allergic inflammation in isolated vagal sensory ganglia unmasks silent NK-2 tachykinin receptors. J. Neurosci. 17, 7683–7693. 9315890 10.1523/JNEUROSCI.17-20-07683.1997 PMC6793896

[B133] Wu Q. Clark M. S. Palmiter R. D. (2012). Deciphering a neuronal circuit that mediates appetite. Nature 483, 594–597. 10.1038/nature10899 22419158 PMC4000532

[B134] Wu X. Y. Zhu J. X. Gao J. Owyang C. Li Y. (2005). Neurochemical phenotype of vagal afferent neurons activated to express C-FOS in response to luminal stimulation in the rat. Neuroscience 130, 757–767. 10.1016/j.neuroscience.2004.09.060 15590158

[B135] Yan X. J. Feng C. C. Liu Q. Zhang L. Y. Dong X. Liu Z. L.. (2014). Vagal afferents mediate antinociception of estrogen in a rat model of visceral pain: the involvement of intestinal mucosal mast cells and 5-hydroxytryptamine 3 signaling. J. Pain 15, 204–217. 10.1016/j.jpain.2013.10.012 24231720

[B136] Zhou X. Galligan J. J. (1999). Synaptic activation and properties of 5-hydroxytryptamine(3) receptors in myenteric neurons of guinea pig intestine. J. Pharmacol. Exp. Ther. 290, 803–810. 10411595

[B137] Zhu J. X. Wu X. Y. Owyang C. Li Y. (2001). Intestinal serotonin acts as a paracrine substance to mediate vagal signal transmission evoked by luminal factors in the rat. J. Physiol. 530, 431–442. 10.1111/j.1469-7793.2001.0431k.x 11158274 PMC2278417

[B138] Zhuo H. Ichikawa H. Helke C. J. (1997). Neurochemistry of the nodose ganglion. Prog. Neurobiol. 52, 79–107. 10.1016/S0301-0082(97)00003-8 9185234

